# Comparison of Growth Inhibition and Clonogenic Assays for Assessing Radiotherapy Responses in Breast Cancer Cell Lines

**DOI:** 10.3390/cancers18111777

**Published:** 2026-05-29

**Authors:** MacKenzie R. Coon-Haworth, P. Finley Durham, Matthew L. Scarpelli

**Affiliations:** School of Health Sciences, Purdue University, West Lafayette, IN 47907, USA

**Keywords:** breast cancer, cell line, clonogenic assay, growth inhibition assay, radiation

## Abstract

**Background**: In vitro assays are widely used to optimize emerging radiotherapy strategies before undertaking more resource-intensive in vivo or clinical studies. However, multiple assay types are currently employed, and it remains unclear whether they yield comparable or complementary measures of radiosensitivity, complicating cross-study comparisons. **Purpose**: This study provides the first direct comparison of two of the most widely used radiosensitivity assays, clonogenic survival and growth inhibition, using commonly reported but non-identical protocols. The goals are to determine whether the assay measurements are correlated and assess their repeatability in breast cancer cell lines. **Methods**: Five cancer cell lines (4T1, SKBR3, BT474, MCF7, and MDA-MB-231) underwent clonogenic and growth inhibition assays following X-ray irradiation at 2, 4, 6, and 8 Gy. Correlations between assay measurements were assessed using Spearman correlations. The entire experiment was repeated twice for each assay to assess repeatability. **Results**: At a given radiation dose, no significant correlations were observed between the clonogenic assay measures and the growth inhibition measures. Across cell lines, the radiation dose resulting in 50% growth inhibition for the growth inhibition assay (mean = 3.2 Gy) was significantly higher than the dose resulting in 50% survival for the clonogenic assay (mean 1.3 Gy; *p* = 0.02). The coefficient of variation between repeat percent of control measurements for growth inhibition assay was trending lower than for clonogenic assay in every experimental condition and was statistically significant at 8 Gy (*p* = 0.02). However, the coefficient of variation values for D_50_ measurements were not significantly different between the assays. **Conclusions**: This procedural comparison demonstrates that clonogenic survival and growth inhibition assays do not yield correlated measurements under the specific conditions tested and instead provide complementary information about cellular radiation response. These findings highlight important practical considerations for assay selection and suggest that the combined use of both assays may provide a more complete characterization of radiosensitivity.

## Simple Summary

Laboratory assays are commonly used to measure how sensitive cancer cells are to radiation before moving to animal or clinical studies. Two of the most widely used assays—clonogenic survival and growth inhibition—are often reported as endpoints, even though they measure different aspects of cell response. In this study, we directly compared these two assays using five breast cancer cell lines exposed to increasing doses of radiation. We found that measurements from the two assays were not correlated at the same radiation dose. This indicates that, under the specific assay conditions tested, the assays capture distinct responses to radiation. Growth inhibition required a higher radiation dose to reduce cell growth by 50% than the dose needed to reduce survival by 50% in clonogenic assays. Together, these findings demonstrate that clonogenic survival and growth inhibition assays provide complementary, rather than equivalent, information. Using both assays may therefore offer a more complete assessment of cellular radiosensitivity.

## 1. Introduction

In vitro assays are widely used to optimize emerging radiotherapy strategies before undertaking more resource-intensive in vivo or clinical studies. However, multiple assay types are currently employed, and it remains unclear whether they yield comparable or complementary measures of radiosensitivity, complicating cross-study comparisons. One of the most common assays used for this purpose is the clonogenic assay developed by Puck and Marcus [1,2,3,4,5,6,7,8,9,10]. Its defining characteristic involves tracking a cell’s ability to form colonies after exposure to various agents [1] (Chapter 3) and [5,8,10]. While it is considered the gold standard in assessing the toxicity of radiation, the clonogenic assay comes with some limitations including long waiting times for single-cell growth, requirement of generating single-cell suspensions, and the lack of consideration for cell-to-cell communication. This is undesirable for studies that wish to conduct numerous experiments as the cost of upkeep and time of a clonogenic assay can quickly compound [8]. As an alternative, some studies have utilized the growth inhibition assay for characterizing radiation responses. This assay tracks the short-term changes in cell number after exposure and can be completed within a few days, offering a faster and simpler approach than the traditional clonogenic assay [9,11,12]. However, it also has some noted limitations, including the potential inclusion of senescent cells in the assay measurements. These cells, although metabolically active, may no longer have the capacity to proliferate. This may not be desired if reproductive cell death is the primary endpoint of interest.

Despite widespread use of both clonogenic and growth inhibition assays, direct comparisons between them are limited and none are focused on breast cancer cell lines. This is a noteworthy gap given that breast cancer is the most common cancer worldwide and often treated with radiotherapy [13,14]. The lack of comparison between growth inhibition and clonogenic assays in breast cancer cell lines is problematic because it is unknown whether the simpler variations of the growth inhibition assay can be used in place of the more labor- and time-intensive clonogenic assay.

The objective of this study was to compare the two procedures side-by-side in mammary and breast cancer cell lines. The comparison includes a direct correlation between the assay measurements, using commonly reported but non-identical protocols. Our initial hypothesis was that the two assays would provide correlated measurements. However, the results suggest the opposite is true with each assay providing a distinct, uncorrelated, measure of cell radiosensitivity. This lack of correlation is important as it suggests the assays should not be used interchangeably or directly compared across studies. To further gain insight into the two assays, we discuss assay tradeoffs regarding ease of use, measurement correlation, and measurement repeatability.

## 2. Methods

### 2.1. Cell Culture and Irradiation

Cell culture and propagation were performed in unison for both assays to maintain comparable conditions. Five cell lines were purchased from American Type Culture Collection (ATCC, Manassas, VA, USA) and passages below 20 were used in these experiments: 4T1 (ATCC, CRL-2539), SKBR3 (ATCC, HTB-30), BT474 (ATCC, HTB-20), MDA-MB-231 (ATCC, CRM-HTB-26), and MCF7 (ATCC, HTB-22). Cell lines 4T1, SKBR3, and BT474 were supplemented with RPMI-1640 (Corning Inc., Corning, NY, USA; 10-041-CV), 10% fetal bovine serum (FBS, Corning, 35-010-CV), and 1% penicillin streptomycin solution (p/s, Corning, 30-002-CI). MDA-MB-231 and MCF7 were supplemented with DMEM (Corning, 10-013-CV), 10% FBS, and 1% p/s. Cells were grown in T-75 flasks (Corning, 430641U) until reaching 70–80% confluency, and then were seeded in accordance with each assay’s protocol.

Cells were irradiated at 0, 2, 4, 6, and 8 Gy using an X-Rad 320 preclinical cabinet irradiator (Precision X-Ray Inc., Madison, CT, USA) with peak energy set to 320 kV, current at 12.5 mA, and beam filtration of 2 mm aluminum yielding a dose rate of 1.8 Gy/min. Machine output was verified monthly using a calibrated ion chamber.

### 2.2. Clonogenic Assay

The clonogenic assay used here is based on the original method of Puck and Marcus and the method described by Franken et al. [2,3,4,5,10]. Before irradiation, cells from T-75 flasks were seeded into T-25 flasks (Corning, 430168) at a seeding density between 10,000 and 50,000 cells/cm^2^, dependent on cell line, one for each dose level. Each T-25 flask was incubated for 24 h at 37 °C and 5% CO_2_ before irradiation. Immediately after irradiation, the cells were passaged into 6-well plates (Corning, 353046) at a low seeding density that increased with dose to accommodate for the escalation in cell death at higher doses, as is customary in clonogenic assay protocols (Supplementary Table S1) [2,5].

Two 6-well plates were prepared to generate 12 clonogenic assay measurements per dose level. The measurements at each dose level were summarized using the mean and standard deviation. The plates were incubated for at least 6 doubling times or until countable colonies were visible in the control (0 Gy) plate. Medium was refreshed every 3–5 days by removing half of the old medium and adding new medium to minimize any disturbance in the growing colonies. The incubation and doubling times for cell lines used are listed in Supplementary Table S1.

For staining, each well was rinsed with phosphate-buffered saline (Corning, 21-040-CV), fixed with 75% methanol (Thermo Fisher Scientific, Waltham, MA, USA) and 25% glacial acetic acid (Thermo Fisher Scientific) for at least 15 min, and then stained with 0.25% crystal violet (Thermo Fisher Scientific) in 20% methanol and 80% water for at least 30 min. After staining, each well was rinsed and set out to dry for at least a day before the colonies were counted.

A colony is defined as containing at least 50 cells. Plates were scanned using a photo scanner (Epson Perfection V600) to compile all the clonogenic assay data and aid in counting. Manual counting was performed to quantify the number of colonies and blinding was not applied. The plating efficiency (PE) and survival fraction (SF) equations are reproduced from multiple established sources [1] (pp. 36–37) and [5,10]. Equation (1) was used to find the control (0 Gy) group’s PE, which was then used in the SF calculation for each dose level using Equation (2). To directly compare the clonogenic and growth inhibition assay results, SFs were converted to percent survival (percent of control) via Equation (3).

(1) $$Plating\;Efficiency\;\left(PE\right)\;=\;\frac{\#\;colonies\;formed\;(untreated;\;0\;Gy)}{\#\;cells\;seeded},$$

(2) $$Survival\;Fraction\;\left(SF\right)=\frac{\#\;colonies\;formed\;(post\;radiation;\;2,\;4,\;6,\;8\;Gy)\;}{\#\;cells\;seeded\times PE},$$

(3) $$Percent\;Survival\left(Percent\;of\;Control\right)=Survival\;Fraction\;\left(SF\right)\times 100\%,$$

### 2.3. Growth Inhibition

For the growth inhibition assay, cells were seeded into five 12-well plates (Corning, 353043) with cell densities between 3000 and 20,000 cells/cm^2^ depending on cell line and incubated at 37 °C and 5% CO_2_ for 24 h. Plates were irradiated at 0, 2, 4, 6, or 8 Gy and then returned to the incubator. Cells were counted at three timepoints, either 24 or 48 h apart depending on the doubling time of each cell line. Log phase growth was verified in the control (0 Gy) plate at all measurement timepoints. At each dose point, two measurements were taken per well across three wells to produce six measurements per dose point. The measurements at each dose point were summarized using the mean and standard deviation.

For cell counting, the supernatant medium was removed, and cells were washed with 0.5 mL of phosphate-buffered saline (Corning, 21-040-CV) per well and then 0.3 mL of trypsin (Corning, 25-052-CI) for 4–5 min. After this, 1 mL of complete medium was added per well to quench the trypsin. Cell suspensions were transferred to Eppendorf tubes (Corning, MCT150C) and thoroughly agitated to form a suspension, and 10 μL of the cell suspension was placed into a hemocytometer for counting.

Three methods of summarizing the growth inhibition assay measurements were evaluated, including (1) calculating a linear fit to the cell count measurements across the timepoints, (2) calculating an exponential fit to the cell count measurements across the timepoints, and (3) using the raw cell count measurements at the final measurement timepoint. None of these methods resulted in significantly correlated measurements with the clonogenic assay and thus, the raw cell counts at the final measurement timepoint were utilized going forward as this was simplest. Equations (4) and (5) describe how the reported numbers were calculated for the growth inhibition assay.

(4) $$Growth\;Inhibition\;Fraction=\frac{\#\;of\;cells\;counted\;(post\;radiation;\;2,\;4,\;6,\;8\;Gy)}{\#\;of\;cells\;counted\;in\;control\;(0\;Gy)},$$

(5) Growth Inhibition Percentage of Control=Growth Inhibition Fraction×100%,

### 2.4. Assay Comparison and Statistical Analysis

The data across dose points was fitted with an exponential, and the D_50_, the dose resulting in 50% survival (clonogenic assay) or growth inhibition (growth inhibition assay) relative to the control, was calculated using the fit. The exponential fit was performed using Python’s (v3.14.5) curve_fit() function (with no weighting) and goodness of fit was assessed by visual inspection (see Supplementary Figures S1 and S2) and R^2^ values (all above 0.93). Correlations between assay percent of control measurements and D_50_ values were assessed using Spearman correlations. Significant differences in assay measurements were assessed using paired two-tailed *t*-tests. The data was log-transformed prior to conducting *t*-tests to meet the normality assumptions of the *t*-test. After transformation, normality was verified by using the Shapiro–Wilk test.

**Figure 1:**
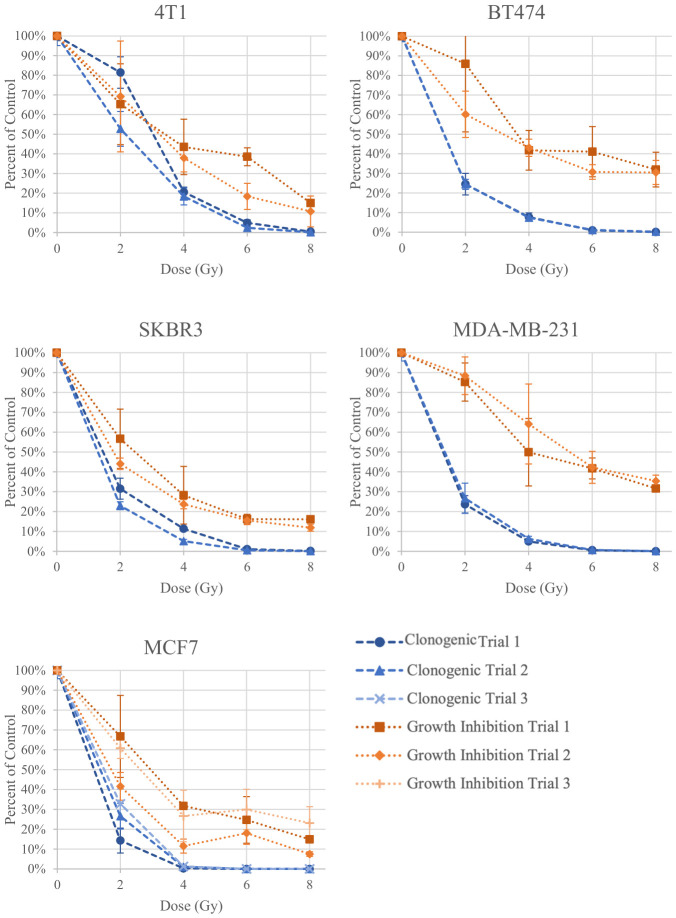
Mean percent of control measurement of each cell line resulting from growth inhibition (orange) and clonogenic assays (blue). Each data point represents the mean and error bars are one standard deviation (*n* = 6 for growth inhibition assay; *n* = 12 for clonogenic assay). To assess repeatability, the entire procedure was repeated twice for each cell line, indicated by the different trials. The procedure was repeated three times for the MCF7 cell line only to verify the relatively low survival values observed at 4 Gy and higher for the clonogenic assay. The legend specifies the assay type and trial number.

The entire experiment described above was repeated twice for each assay to assess repeatability. Measurement for the MCF7 cell line only was repeated a third time to confirm the low clonogenic survival observed at higher doses (see Figure 1). Repeatability was quantified by calculating the coefficient of variation (CV) between measurements from the repeated experiments [15,16,17,18,19].

## 3. Results

### 3.1. Comparison of Growth Inhibition and Clonogenic Assay Measurements

Representative images of colony formation and growth inhibitions are found in Supplementary Figures S3 and S4, respectively. Clonogenic assay images illustrate clear differences in colony size and morphology across cell lines. As expected, higher radiation doses resulted in lower percent of control measurements for both assays. The clonogenic assay was particularly sensitive to radiation effects with some cell lines at nearly 0% clonogenic survival at 6 and 8 Gy (Figure 1).

**Figure 2:**
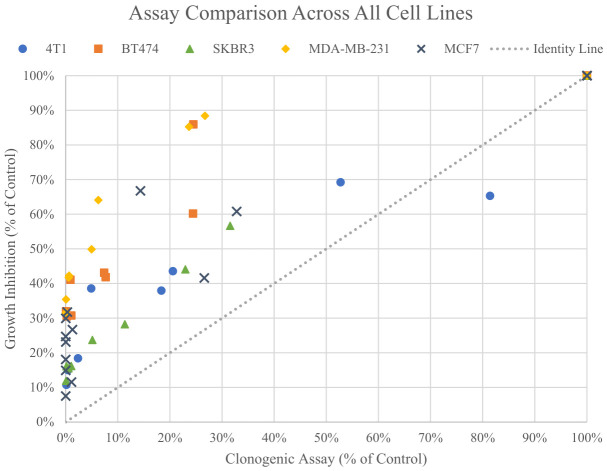
Scatter plot of mean percent of control measurement between growth inhibition (vertical axis) and clonogenic assay (horizontal axis). Results for Trials 1, 2, and 3 are included. Each data point represents the mean of 6 (growth inhibition) or 12 (clonogenic) measurements. Identity line added to visualize trends.

Overall, a positive association was observed between growth inhibition and clonogenic measurements (Figure 2). Most data points are above the identity line, indicating the growth inhibition assay measured higher percent of control values than the clonogenic assay. This was further verified when sub-dividing the data by radiation dose, with the mean percent of control values being significantly higher for growth inhibition assay (counts at final timepoint) than clonogenic assay at every dose level (Table 1). The correlation between assay measurements was not statistically significant at any dose level (Table 1). The correlation between the clonogenic assay measurements and the other growth inhibition assay techniques (e.g., linear fit and exponential fit across timepoints) are included in Supplementary Tables S2 and S3. No significant correlation was identified between any of the growth inhibition assay techniques and clonogenic assay measurements.

The mean D_50_ estimates ranged from the lowest mean value of 0.96 Gy in MCF7 measured using the clonogenic assay to the highest mean value of 5.30 Gy in MDA-MB-231 measured using the growth inhibition assay (Table 2). Across cell lines, the growth inhibition assay (counts at final timepoint) produced a significantly higher mean D_50_ value (3.21 Gy) than the clonogenic assay (1.26 Gy) (*p* = 0.02). The Spearman correlation between the assay D_50_ values was −0.10 (*p* = 0.87), indicating little to no association.

### 3.2. Assay Repeatability

The mean CV values increased with dose for both assays (Table 3, Table 4 and Table 5). Table 3 shows the CV values across the well plate measurements within Trial 1 (the values for Trial 2 are shown in Supplementary Table S4). The mean CV for the well plate measurements differed significantly between the assays at 8 Gy, with growth inhibition displaying lower CV values (*p* = 0.04). Table 4 shows the CV values of the percent of control measurement across the trials. For clonogenic assays, the mean CV for percent of control measurements increased from a mean value of 0.20 at 2 Gy to 0.84 at 8 Gy, whereas growth inhibition increased from a mean value of 0.15 at 2 Gy to 0.21 at 8 Gy. The mean CV for percent of control measurements differed significantly between assays at 8 Gy, with growth inhibition displaying lower CV values (*p* = 0.02). No significant differences were observed between assay CV values at the other dose levels. CVs for the D_50_ estimates across trials are provided in Table 5. The D_50_ mean CV value was 0.15 for the clonogenic assay and 0.21 for the growth inhibition assay. These D_50_ mean CV values were not significantly different between assays (*p* = 0.39). The full dataset used in our analyses can be found in Supplementary Tables S5 and S6.

## 4. Discussion

The clonogenic assay is the gold standard for assessing radiosensitivity because it captures all forms of reproductive cell death, but it is time- and resource-intensive [5,8,9,20]. The growth inhibition assay offers a potentially simpler, faster alternative, avoiding the complexities of colony formation assays [9]. Although both assays have been used to measure radiosensitivity, they measure two different but related endpoints. In the case of clonogenic assay, the measurements quantify clonogenic survival and in the case of growth inhibition assay, the measurements quantify cell growth. This study aimed to determine whether growth inhibition measurements correlate with those of the clonogenic assay and whether it could serve as a practical substitute when resources or throughput needs are limited. This is the first time these specific assays have been directly compared by correlation, providing insight into the relationship between the two assays.

As expected, both assays’ measurements decreased with increasing radiation dose, indicating reduced cell growth and survival. However, when comparing assay measurements at specific dose levels, there was no statistically significant correlation. This suggests the two assays measure distinct aspects of cellular radiosensitivity, under the specific conditions tested. In addition, the growth inhibition assay consistently measured higher percent of control values than the clonogenic assay at a given radiation dose level. This may reflect the fact that the cells for the clonogenic assay were replated immediately after irradiation, which diminishes the DNA repair capacity of the cells. Performing the replating for clonogenic assay prior to irradiation may improve the agreement between the two assay measurements (Supplementary Figure S5). Cell density may also influence the assay measurements as the clonogenic assay was seeded at 10–50 thousand cells/cm^2^ and growth inhibition was seeded at 3–20 thousand cells/cm^2^ for irradiation. In addition, the clonogenic assay’s low seeding density following single-cell suspension decreases cell-to-cell communication [12,21,22,23]. The shorter follow-up time of the growth inhibition assay relative to the clonogenic assay may prevent measurement of radiation effects occurring over a longer timescale, which could partially explain the higher percent of control measurement for the growth inhibition assay. These methodological differences may account for the lack of agreement between assay measurements in the current study. This lack of agreement suggests these two assay cannot be used interchangeably with the methods outlined in this study and reported in prior literature. These findings motivate future work to identify methodological steps to give better agreement between the two assays. This would enable determination of whether or not a ‘simplified’ version of growth inhibition assay may be used in place of the more time-consuming clonogenic assay. One limitation of this work was the lack of inclusion of a non-cancerous breast epithelial cell line, which could have provided insight on whether the observed assay differences are specific to cancer cells or reflect general biological properties of mammary cells.

The observed variation in D_50_ values of the current study suggests a wide range of radiosensitivity across the cell lines. This may result from differences in cell line apoptosis, DNA repair, and/or cell cycle times [24]. MCF7 demonstrated relatively low survival for clonogenic assay at higher radiation doses. This unusual observation could be due to technical artifacts such as incomplete single-cell dissociation or clump formation, rather than true biologic radiosensitivity. Further follow-up to identify specific mechanisms underlying the cell-line-specific variations in radiosensitivity could allow for insight into the variable clinical responses observed in breast cancer radiotherapy [25,26,27].

Repeatability of the clonogenic and growth inhibition assays was quantified using the CV values as in other repeatability studies [8,15,16,17]. The clonogenic assay showed higher CV values than the growth inhibition assay, which was statistically significant at the 8 Gy dose level for the percent of control measurements. This CV difference between assays could be partially attributed to the fact that there were few surviving colonies measured at 8 Gy for the clonogenic assay. The low number of surviving colonies increases the relative fluctuations in the percent of control measurements due to division by a near-zero number. This does not indicate poor physical repeatability but rather a mathematical artifact of the calculation. In addition, the challenges of creating quality single-cell suspensions might also contribute to increased variability in clonogenic assay measurements [9,11]. The CV of the D_50_ measurements did not differ significantly between the assays. This suggests D_50_ may be a more repeatable radiosensitivity metric than assay measurements at a single dose level, especially at high dose levels where there are few surviving colonies.

The general assumption when doing clonogenic assay is that the plating efficiency maintains a linear relationship between the seeding density and the resultant colonies for untreated cells [8,9]. These assumptions may not hold in all cases and are a limitation of using the clonogenic assay for some cell lines [8,11]. In addition, the challenges of generating single-cell seeding densities can add additional uncertainty in clonogenic assay measurements. The growth inhibition assay also has limitations. For example, the growth inhibition assay measurements may not be as sensitive to low radiation doses as clonogenic assay measurements. In addition, the growth inhibition assay includes senescent and non-dividing cells, which may not be desired if aiming to measure reproductive cell death. The growth inhibition assay is also typically performed over a much shorter timescale than the clonogenic assay, which may miss any radiation effects that take place over longer timescales. Thus, the choice of which assay to utilize will depend on careful consideration of the strengths and limitations of each assay and the desired endpoint. If resources are limited and a high-throughput assay is desired, the growth inhibition assay may be the preferred option. It can be performed more quickly than the clonogenic assay and does not require the generation of a single-cell suspension, which can be challenging for some cell lines. However, if a more rigorous assessment of reproductive survival is desired then the clonogenic assay would be the ideal choice. It remains the gold standard for assessing all forms of reproductive cell death. Given the lack of correlation between assay measurements, utilization of both assays might be warranted in some cases as each assay provides distinct information regarding radiosensitivity. If the assays provide discordant results, then follow-up using senescence markers or assessing cell signaling molecules could provide insight [28,29,30]. In addition, comparison of the assay measurements with in vivo measures of tumor radiosensitivity may provide further insight into which assay is the best surrogate of in vivo responses.

## 5. Conclusions

This study provides a direct comparison between the growth inhibition and clonogenic assays, using commonly reported but non-identical protocols. The results indicated the assay measurements differed significantly and were uncorrelated, indicating the assays cannot substitute for one another or be compared across studies. Rather, our results suggest the assays, under the specific conditions tested, offer complementary measures of radiosensitivity and utilization of both assays may be warranted for a more comprehensive assessment of cell radiosensitivity. Future studies could identify methodological steps to give better agreement between the two assays. This would enable determination of whether or not a ‘simplified’ version of growth inhibition assay may be used in place of the more time-consuming clonogenic assay.

## Figures and Tables

**Table 1:** Mean percent of control and standard deviation values across cell lines (*n* = 5 per dose level).

PC *	Growth Inhibition Mean	Growth Inhibition Standard Deviation	Clonogenic Mean	Clonogenic Standard Deviation	*t*-Test *p*-Value **	Spearman Rho (*p*-Value)
PC_2_	66.8%	14.3%	33.7%	18.7%	0.03	−0.30 (0.62)
PC_4_	37.9%	13.7%	8.4%	6.8%	0.01	0.10 (0.87)
PC_6_	29.3%	10.1%	1.2%	1.4%	0.02	−0.15 (0.81)
PC_8_	21.4%	10.1%	0.1%	0.1%	0.002	−0.50 (0.39)

* PC percent of control (relative to 0 Gy) for each dose, i.e., 2, 4, 6, and 8 Gy. ** The *t*-test tests the null hypothesis that the mean value of growth inhibition assay is significantly different than the mean value of the clonogenic assay.

**Table 2:** D_50_ values for growth inhibition and clonogenic assays. The bold D_50_ values are the mean values across Trials for each cell line.

Cell Line	Growth Inhibition	Clonogenic Assay
4T1 Trial 1	3.58	2.83
4T1 Trial 2	3.12	1.96
**4T1 Mean**	**3.35**	**2.39**
BT474 Trial 1	4.48	1.00
BT474 Trial 2	2.93	1.00
**BT474 Mean**	**3.70**	**1.00**
SKBR3 Trial 1	2.24	1.23
SKBR3 Trial 2	1.69	0.95
**SKBR3 Mean**	**1.97**	**1.09**
MDA-MB-231 Trial 1	4.87	0.96
MDA-MB-231 Trial 2	5.73	1.05
**MDA-MB-231 Mean**	**5.30**	**1.01**
MCF7 Trial 1	2.86	0.71
MCF7 Trial 2	1.44	1.01
MCF7 Trial 3	2.41	1.16
**MCF7 Mean**	**2.24**	**0.96**
**Overall Mean**	**3.21**	**1.26**

**Table 3:** Coefficient of variation (CV) values of well plate measurements in Trial 1 (*n* = 12 wells for clonogenic assay; *n* = 6 wells for growth inhibition assay).

Cell Line	4T1		BT474		SKBR3		MDA-MB-231		MCF7		Mean		*p*-Values ^3^
Dose (Gy)	Clon ^1^	GI ^2^	Clon	GI	Clon	GI	Clon	GI	Clon	GI	Clon	GI	
2	0.1	0.35	0.22	0.39	0.17	0.12	0.19	0.16	0.44	0.2	0.22	0.24	0.79
4	0.12	0.23	0.28	0.22	0.09	0.39	0.19	0.35	1.15	0.18	0.37	0.27	0.83
6	0.32	0.19	0.46	0.29	0.37	0.24	0.7	0.08	3.46	0.44	1.06	0.25	0.05
8	0.48	0.28	0.6	0.26	0.51	0.14	1.48	0.08	3.46	0.09	1.31	0.17	0.04
Mean	0.26	0.26	0.39	0.29	0.29	0.22	0.64	0.17	2.13	0.23			
*p*-values ^4^	0.67		0.48		0.81		0.26		0.04				

^1^ Clon, clonogenic assay, ^2^ GI, growth inhibition assay. ^3^ *p*-value comparing CV values within a given dose level, ^4^ *p*-values comparing CV values within a given cell line.

**Table 4:** Coefficient of variation (CV) values of percent of control measurements repeated across trials (*n* = 2 trials).

Cell Line	4T1		BT474		SKBR3		MDA-MB-231		MCF7		Mean		*p*-Values ^3^
Dose (Gy)	Clon ^1^	GI ^2^	Clon	GI	Clon	GI	Clon	GI	Clon	GI	Clon	GI	
2	0.30	0.04	0.002	0.25	0.22	0.18	0.09	0.03	0.38	0.23	0.20	0.15	0.90
4	0.08	0.10	0.03	0.02	0.53	0.12	0.16	0.18	0.57	0.45	0.28	0.17	0.33
6	0.49	0.50	0.13	0.20	0.48	0.03	0.08	0.01	1.73	0.25	0.58	0.20	0.12
8	0.72	0.23	0.69	0.03	0.45	0.21	0.61	0.08	1.73	0.51	0.84	0.21	0.02
Mean	0.40	0.22	0.21	0.13	0.42	0.14	0.23	0.07	1.11	0.36			
*p*-values ^4^	0.25		0.79		0.10		0.08		0.09				

^1^ Clon, clonogenic assay, ^2^ GI, growth inhibition assay. ^3^ *p*-value comparing CV values within a given dose level, ^4^ *p*-values comparing CV values within a given cell line.

**Table 5:** Coefficient of variation (CV) of D_50_ values repeated across trials (*n* = 2 trials).

Cell Line	4T1	BT474	SKBR3	MDA-MB-231	MCF7	Mean	*t*-Test *p*-Value
Clonogenic Assay	0.26	4.33 × 10^−3^	0.18	0.06	0.24	0.15	0.39
Growth Inhibition	0.10	0.30	0.20	0.11	0.32	0.21	

## Data Availability

Please see the Supplementary Materials for data used in the manuscript. In addition, all data will be shared upon reasonable request to the corresponding author.

## Supplementary Materials

The following supporting information can be downloaded at https://www.mdpi.com/article/10.3390/cancers18111777/s1. Figure S1: Growth inhibition exponential fits for calculation of D_50_ values (each data point represents the mean value of 6 technical replicates). Figure S2: Clonogenic assay exponential fits for calculating D_50_ values (each data point represents the mean value of 12 technical replicates). Figure S3: Images of the resultant clonogenic assay after staining of different cell lines and dose points. Figure S4: Representative images of the modified growth inhibition assay counts utilizing a hemocytometer for cell line SKBR3. Figure S5: Comparison of growth inhibition assay with cell seeding prior to irradiation (no replating) versus replating cells immediately following irradiation. Table S1: Clonogenic assay seeding densities (in cells per well) in a 6-well plate. Table S2: Growth inhibition assay values with a linear fit to measurement timepoints and summarized across cell lines (*n* = 5 per dose level). Table S3: Growth inhibition assay values with a logarithmic fit to measurement timepoints and summarized across cell lines (*n* = 5 per dose level). Table S4: Coefficient of variation (CV) values of well plate measurements in Trial 2 (*n* = 12 wells for clonogenic assay; *n* = 6 wells for growth inhibition assay). Table S5: Clonogenic assay percent of control values and plating efficiency (PE) values (*n* = 12). Table S6: Growth inhibition assay percent of control values (*n* = 6).
