# Supplementary material for: Comparison of Growth Inhibition and Clonogenic Assays for Assessing Radiotherapy Responses in Breast Cancer Cell Lines
Source: Cancers (Basel). 2026 May 29;18(11):1777. doi: 10.3390/cancers18111777 (PMC13256728; doi:10.3390/cancers18111777)
Supplement: Supplementary file 1 [file cancers-18-01777-s001.zip › cancers-4245502-supplementary.pdf]

Supplementary Figure S1: Growth inhibition exponential fits for calculation of D_{50} values (each data point represents the mean value of 6 technical replicates).

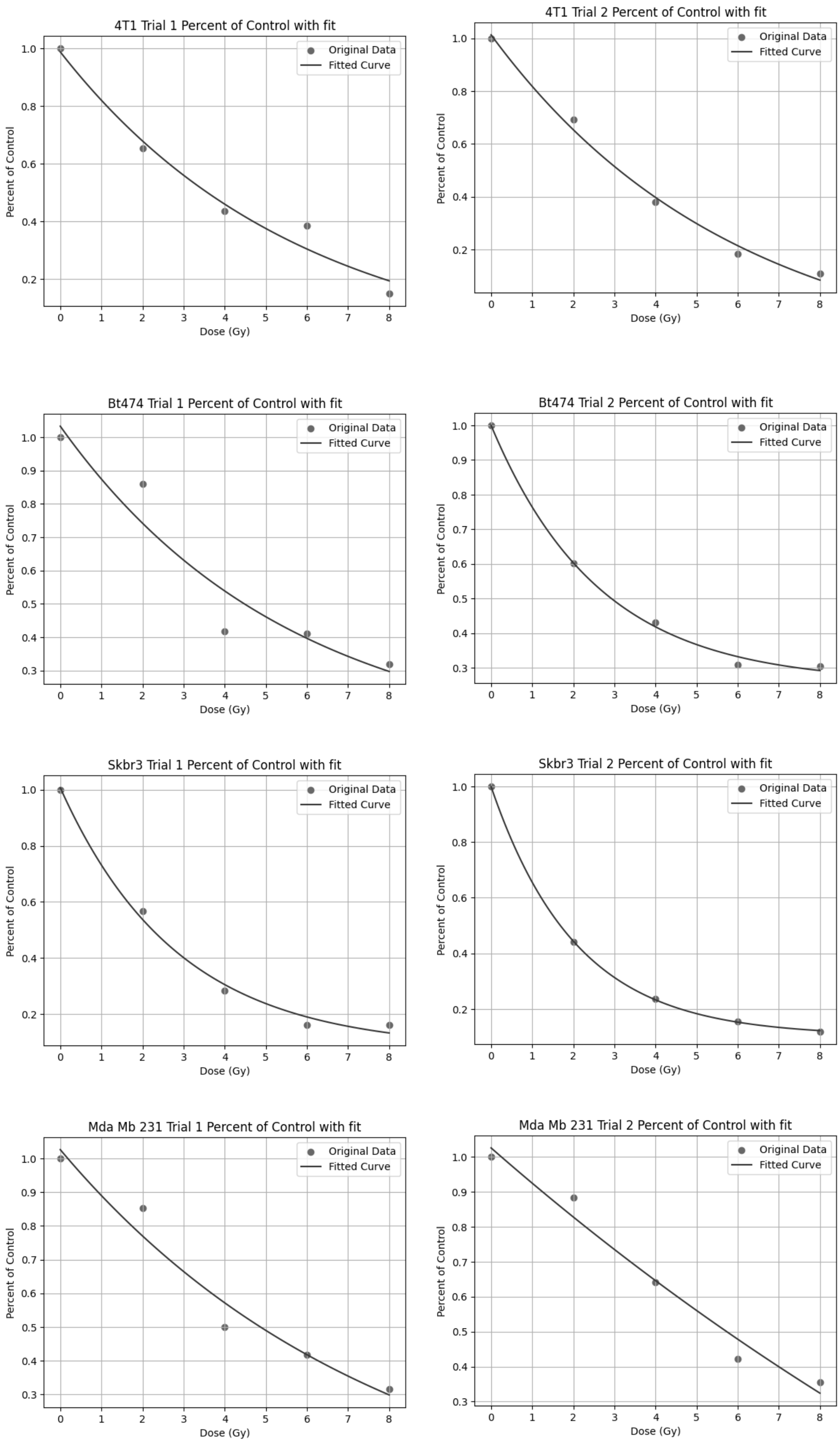

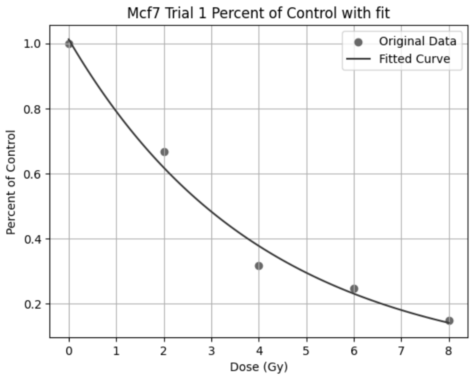
Mcf7 Trial 1 Percent of Control with fit
Original Data
Fitted Curve
Percent of Control
Dose (Gy)
1.0
0.8
0.6
0.4
0.2
0
1
2
3
4
5
6
7
8

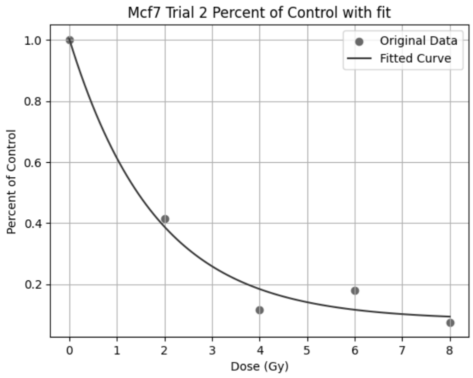
Mcf7 Trial 2 Percent of Control with fit
Original Data
Fitted Curve
Percent of Control
Dose (Gy)
1.0
0.8
0.6
0.4
0.2
0
1
2
3
4
5
6
7
8

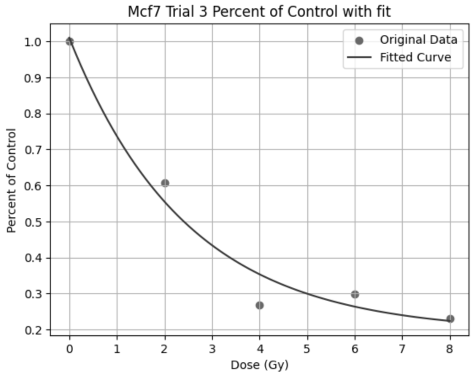
Mcf7 Trial 3 Percent of Control with fit
Original Data
Fitted Curve
Percent of Control
Dose (Gy)
1.0
0.9
0.8
0.7
0.6
0.5
0.4
0.3
0.2
0
1
2
3
4
5
6
7
8

Supplementary Figure S2: Clonogenic assay exponential fits for calculating D_{50} values (each data point represents the mean value of 12 technical replicates).
.

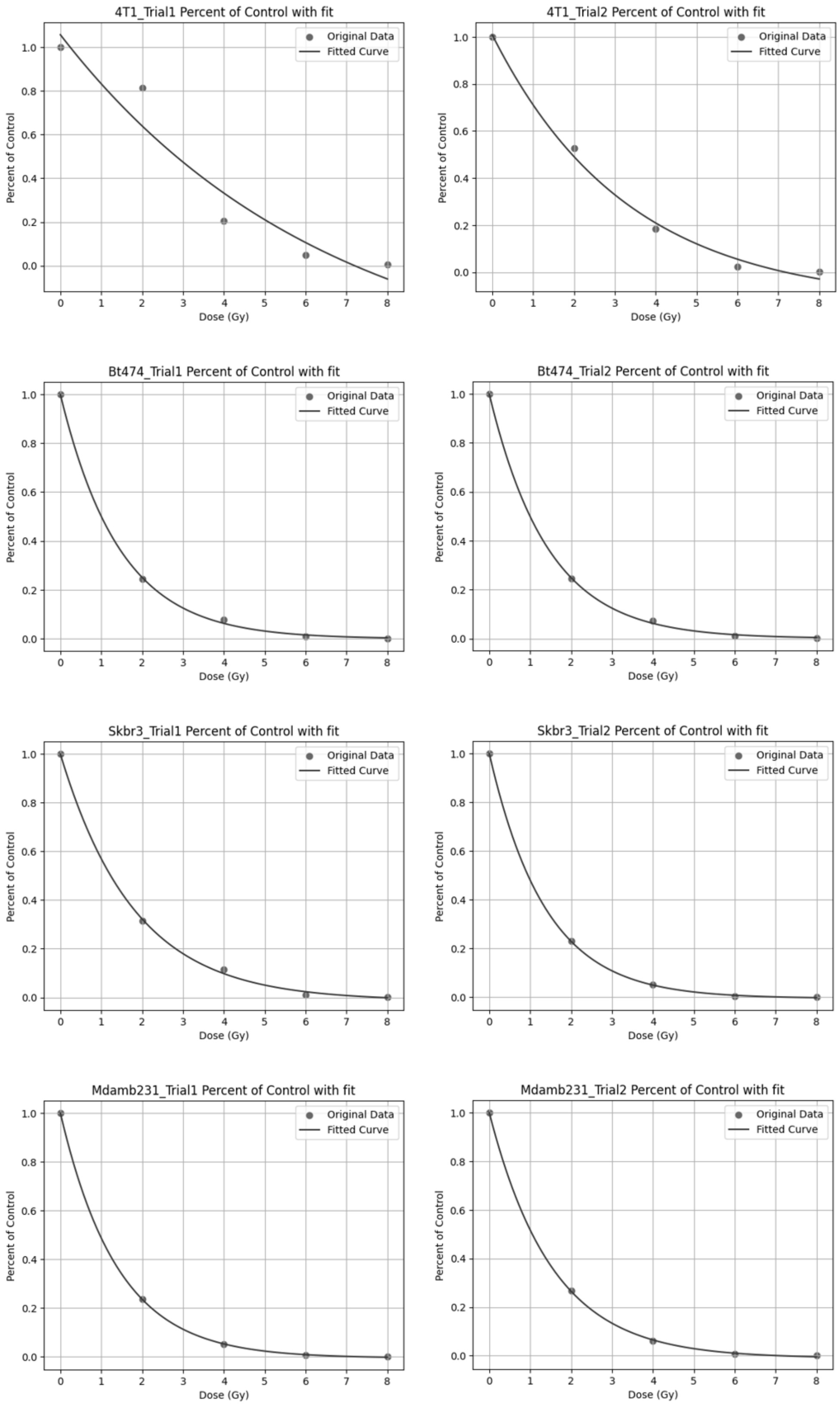

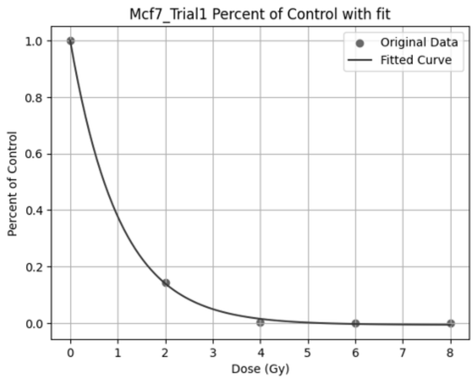
Mcf7_Trial1 Percent of Control with fit
Original Data
Fitted Curve
Percent of Control
Dose (Gy)

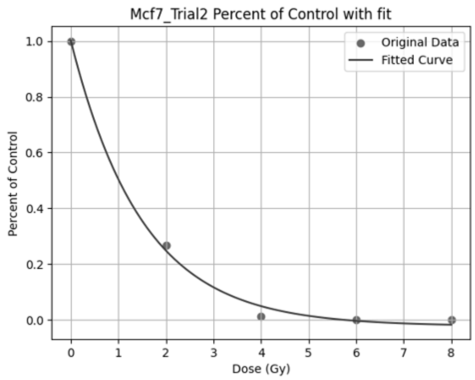
Mcf7_Trial2 Percent of Control with fit
Original Data
Fitted Curve
Percent of Control
Dose (Gy)

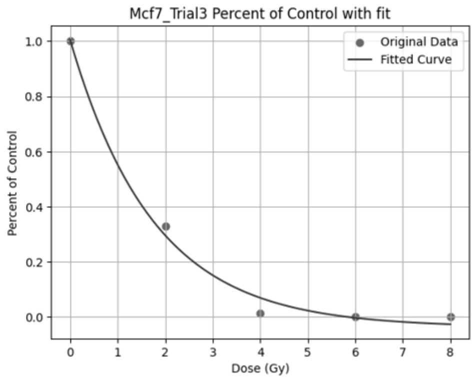
Mcf7_Trial3 Percent of Control with fit
Original Data
Fitted Curve
Percent of Control
Dose (Gy)

Supplementary Figure S3: Images of the resultant clonogenic assay after staining of different cell lines and dose points.

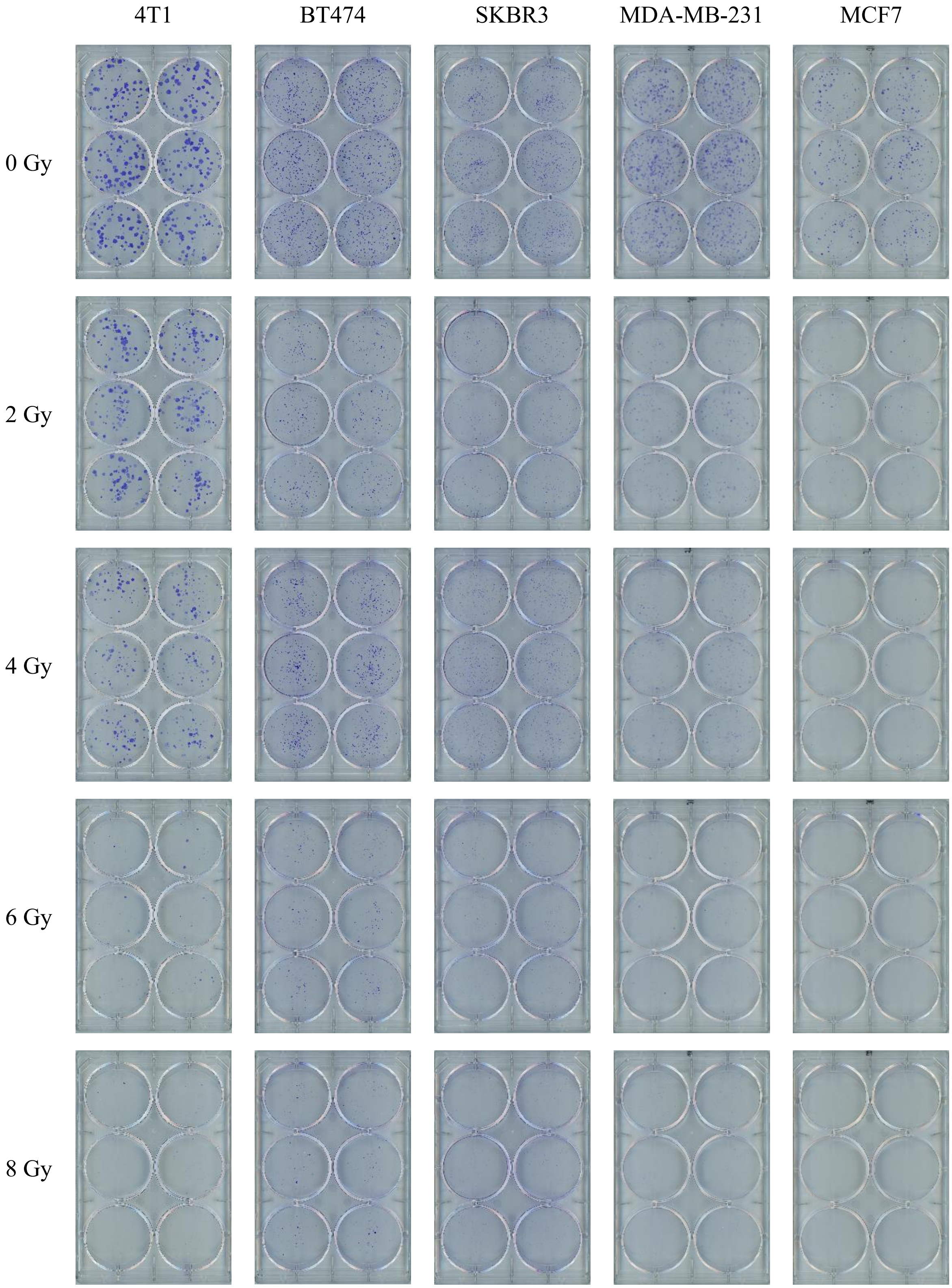

Supplementary Figure S4: Representative images of the modified growth inhibition assay counts utilizing a hemocytometer for cell line SKBR3.

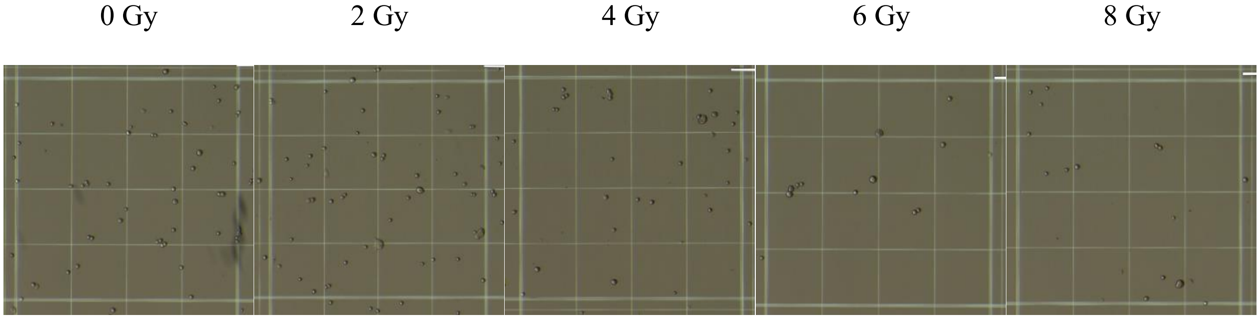

Supplementary Figure S5: Comparison of growth inhibition assay with cell seeding prior to irradiation (no replating) versus replating cells immediately following irradiation. The orange curves are the original growth inhibition data with no replating (Figure 1 of the manuscript). The purple curves are the growth inhibition data with immediate replating following irradiation. The curves for both are relatively close together. In addition, two different initial seeding densities (prior to irradiation) were assessed. One case with an initial seeding density of 10,000 cell/cm^2 (dark purple curve) and one with an initial seeding density of 5,000 cell/cm^2 (light purple curve). For the different initial seeding densities, the curves are relatively close together. This suggests that, for 4T1 at least, the differences between no replate vs immediate replate as well as seeding density (over the range assessed) do not have a large impact on growth inhibition measurements. GI = Growth Inhibition.

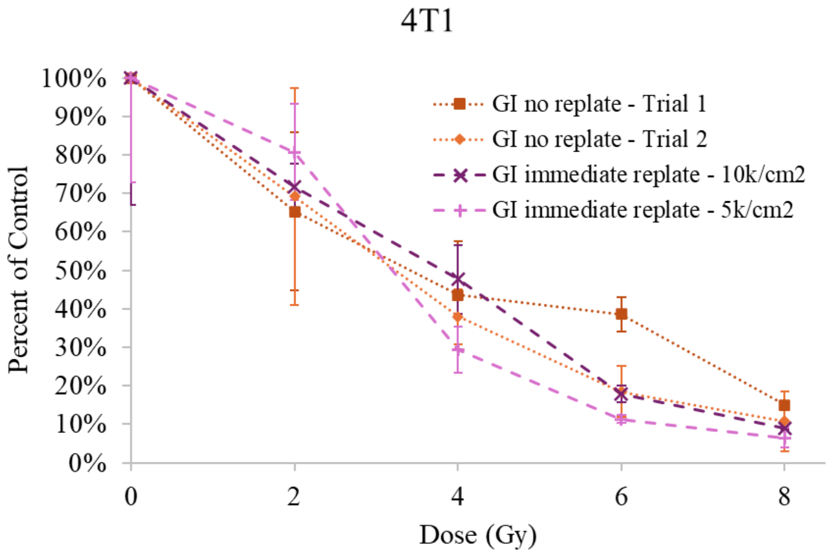

Supplementary Table S1: Clonogenic assay seeding densities (in cells per well) in a 6-well plate.

Cell Line	4T1	BT474	SKBR3	MDA-MB-231	MCF7
**Clonogenic Incubation Time**	**7 days**	**28 days**	**14 days**	**11 days**	**14 days**
**Approximate Doubling Time**	**13 hr**	**60 hr**	**35 hr**	**25 hr**	**35 hr**
**0 Gy**	200	1000	1000	500	500
**2 Gy**	200	1000	1000	500	500
**4 Gy**	800	5000	5000	2000	2000
**6 Gy**	800	5000	5000	2000	2000
**8 Gy**	2000	10000	10000	5000	5000

Supplementary Table S2: Growth inhibition assay values with a linear fit to measurement timepoints and summarized across cell lines (n=5 per dose level).

PC*	Growth Inhibition Mean***	Growth Inhibition Standard Deviation	Clonogenic Mean	Clonogenic Standard Deviation	T-Test** p-value	Spearman Rho (p-value)
**PC_2**	64.6%	18.3%	33.7%	18.7%	0.03	0.10 (0.95)
**PC_4**	21.5%	22.3%	8.4%	6.8%	0.17	0.20 (0.78)
**PC_6**	10.6%	17.2%	1.2%	1.4%	0.04	-0.20 (0.78)
**PC_8**	-2.0%	11.8%	0.1%	0.1%	0.02	0.30 (0.68)

*PC percent of control (relative to 0 Gy) for each dose i.e., 2, 4, 6, and 8 Gy.

** The T-Test tests the null hypothesis that the mean value of growth inhibition assay is significantly different than the mean value of the clonogenic assay.

***Linear Growth Rate $= \frac{(\text{Count @ final timepoint}) - (\text{Count @ initial timepoint})}{\text{final timepoint} - \text{initial timepoint}}$. The linear growth rate at each dose level was divided by the 0 Gy growth rate to get the percent of control values shown.

Supplementary Table S3: Growth inhibition assay values with a logarithmic fit to measurement timepoints and summarized across cell lines (n=5 per dose level).

PC*	Growth Inhibition Mean***	Growth Inhibition Standard Deviation	Clonogenic Assay	Clonogenic Standard Deviation	T-Test** p-value	Spearman Rho (p-value)
**PC_2**	92.8%	14.4%	33.7%	18.7%	0.003	0.50 (0.45)
**PC_4**	50.1%	37.0%	8.4%	6.8%	0.11	0.50 (0.45)
**PC_6**	31.4%	37.0%	1.2%	1.4%	0.02	0.30 (0.68)
**PC_8**	0.0%	35%	0.1%	0.1%	0.01	0.60 (0.35)

*PC percent of control (relative to 0 Gy) for each dose i.e., 2, 4, 6, and 8 Gy.

** The T-Test tests the null hypothesis that the mean value of growth inhibition assay is significantly different than the mean value of the clonogenic assay.

*** Log Growth Rate $= \frac{\ln\left(\text{(Count @ final timepoint)}/\text{(Count @ initial timepoint)}\right)}{\ln(2)}$. The logarithmic growth rate at each dose level was divided by the 0 Gy growth rate to get the percent of control values shown.

Supplementary Table S4: Coefficient of variation (CV) values of well plate measurements in Trial 2 (n=12 wells for clonogenic assay; n=6 wells for growth inhibition assay).

Cell Line	4T1		BT474		SKBR3		MDA-MB-231		MCF7		Mean		p-values[3]
**Dose (Gy)**	**Clon[1]**	**GI[2]**	**Clon**	**GI**	**Clon**	**GI**	**Clon**	**GI**	**Clon**	**GI**	**Clon**	**GI**	
**2**	0.17	0.23	0.11	0.21	0.09	0.01	0.28	0.05	0.23	0.12	**0.18**	**0.12**	**0.26**
**4**	0.23	0.12	0.12	0.1	0.18	0.12	0.21	0.32	0.49	0.36	**0.25**	**0.20**	**0.28**
**6**	0.27	0.1	0.26	0.12	0.39	0.11	0.58	0.27	N/A[5]	0.24	**0.38**	**0.17**	**0.004**
**8**	0.72	0.52	0.41	0.05	0.65	0.09	1.24	0.18	N/A[5]	0.1	**0.76**	**0.19**	**0.03**
**Mean**	**0.35**	**0.24**	**0.23**	**0.12**	**0.33**	**0.08**	**0.58**	**0.21**	**0.36**	**0.21**			
**p-values[4]**	**0.23**		**0.37**		**0.04**		**0.16**		**0.22**				

[1] Clon, clonogenic assay
[2] GI, growth inhibition assay.
[3] p-value comparing CV values within a given dose level
[4] p-values comparing CV values across within a given cell line
[5]No colonies counted at these doses for the clonogenic assay

Supplementary Table S5: Clonogenic assay percent of control values and plating efficiency (PE) values (n = 12).

F_{Dose}	4T1 (%)		BT474 (%)		SKBR3 (%)		MDA-MB-231 (%)		MCF7 (%)		
	**Trial 1**	**Trial 2**	**Trial 1**	**Trial 2**	**Trial 1**	**Trial 2**	**Trial 1**	**Trial 2**	**Trial 1**	**Trial 2**	**Trial 3**
**PE**	**0.39**	**0.50**	**0.28**	**0.28**	**0.24**	**0.26**	**0.25**	**0.25**	**0.12**	**0.19**	**0.19**
F_2	81	53	25	24	32	23	24	27	14	27	33
F_4	21	18	7.7	7.4	11	5.1	5.0	6.3	0.31	1.1	1.3
F_6	4.9	2.4	0.92	1.1	1.1	0.53	0.58	0.65	0	0	0.02
F_8	0.46	0.15	0.08	0.22	0.16	0.08	0.03	0.07	0	0	0.01

Supplementary Table S6: Growth inhibition assay percent of control values (n = 6).

F_{Dose}	4T1 (%)		BT474 (%)		SKBR3 (%)		MDA-MB-231 (%)		MCF7 (%)		
	**Trial 1**	**Trial 2**	**Trial 1**	**Trial 2**	**Trial 1**	**Trial 2**	**Trial 1**	**Trial 2**	**Trial 1**	**Trial 2**	**Trial 3**
F_2	65	69	86	60	57	44	85	88	67	42	61
F_4	44	38	42	43	28	24	50	64	32	12	27
F_6	39	18	41	31	16	16	42	42	25	18	30
F_8	15	11	32	31	16	12	32	35	15	7.5	23
